# Supplementary material for: Adaptation and Validation of the Constructivist Teaching Practices Inventory in Elementary Physical Education (CTPI-EPE) for Brazilian Physical Education Pre-Service Teachers
Source: Int J Environ Res Public Health. 2022 Sep 24;19(19):12091. doi: 10.3390/ijerph191912091 (PMC9564738; doi:10.3390/ijerph191912091)
Supplement: Supplementary file 1 [file ijerph-19-12091-s001.zip › ijerph-1843377-SI.pdf]

Table S1. Standardized residuals covariance matrix.

	I1	I4	I5	I7	I8	I9	I10	I11	I12	I14	I15	I16	I17	I18	I20	I21	I22	I23	I24	I25	I26	I28	I32	I33	I36
I1	0.00																								
I4	0.04	0.00																							
I5	0.04	0.04	0.00																						
I7	-0.04	0.00	-0.04	0.00																					
I8	-0.03	-0.02	0.03	0.04	0.00																				
I9	-0.02	-0.02	0.01	0.05	-0.01	0.00																			
I10	-0.01	0.03	0.01	0.02	-0.02	0.09	0.00																		
I11	-0.02	0.01	-0.01	0.03	-0.00	0.04	0.02	0.00																	
I12	0.10	-0.01	0.01	0.01	-0.04	-0.03	-0.05	0.00	0.00																
I14	-0.03	-0.03	0.03	-0.00	0.07	-0.05	-0.05	0.05	0.01	0.00															
I15	-0.00	0.03	0.01	-0.04	-0.00	-0.04	-0.01	-0.02	0.03	0.01	0.00														
I16	-0.00	0.01	-0.00	0.01	0.02	0.00	0.01	-0.02	-0.01	-0.04	0.04	0.00													
I17	0.05	0.01	0.08	-0.03	0.06	0.04	0.02	0.03	0.11	0.09	0.10	0.09	0.00												
I18	0.02	-0.06	-0.01	-0.01	-0.01	-0.05	-0.05	-0.04	0.05	0.06	0.01	-0.05	0.05	0.00											
I20	0.02	-0.02	-0.03	-0.01	0.02	-0.06	-0.04	-0.04	0.01	0.03	0.01	-0.03	-0.00	0.09	0.00										
I21	0.01	-0.02	-0.06	-0.01	0.03	-0.05	-0.03	-0.04	0.02	0.06	0.02	-0.06	-0.02	0.03	0.10	0.00									
I22	0.04	-0.06	-0.04	0.05	0.05	0.00	-0.05	0.01	0.03	0.09	0.01	-0.03	-0.01	0.03	0.01	0.05	0.00								
I23	0.05	0.02	0.11	0.03	0.04	0.01	-0.00	-0.01	0.05	0.06	0.02	0.03	0.05	-0.04	-0.04	-0.01	-0.00	0.00							
I24	0.06	0.02	0.03	0.06	0.00	0.01	-0.03	0.02	0.06	0.02	0.06	0.10	0.02	-0.06	-0.03	-0.04	-0.04	0.13	0.00						
I25	0.02	-0.02	0.02	0.02	-0.00	0.02	-0.02	0.01	0.04	0.02	0.05	0.03	0.00	-0.06	-0.04	-0.03	-0.01	0.03	0.10	0.00					
I26	0.06	-0.06	0.00	-0.01	-0.00	-0.07	-0.08	-0.03	0.05	0.09	0.01	0.02	0.04	0.03	-0.01	-0.04	0.01	0.00	0.03	0.04	0.00				
I28	0.05	-0.04	-0.07	0.01	0.00	-0.03	-0.06	-0.05	0.01	0.04	0.03	0.03	0.01	-0.00	-0.02	-0.03	0.01	-0.03	0.00	0.03	0.06	0.00			
I32	0.01	0.00	-0.03	-0.01	-0.05	-0.02	-0.02	-0.04	0.03	-0.03	0.02	0.01	-0.04	-0.02	-0.02	-0.03	-0.01	0.00	-0.00	-0.00	-0.01	0.08	0.00		
I33	-0.01	0.06	-0.01	0.06	0.01	0.04	0.10	0.08	0.05	0.01	0.09	0.06	-0.03	-0.06	-0.05	-0.02	-0.03	-0.00	-0.01	0.05	-0.05	-0.02	0.07	0.00	
I36	-0.01	0.00	-0.01	0.03	0.01	-0.00	-0.01	-0.03	0.05	0.00	0.07	0.01	0.01	0.02	-0.00	-0.01	-0.02	-0.04	-0.04	-0.02	-0.02	0.01	0.05	0.07	0.00

	I1	I4	I5	I7	I8	I9	I11	I12	I15	I16	I18	I21	I22	I23	I25	I26	I28	I32	I36
I1	0.00																		
I4	0.04	0.00																	
I5	0.04	0.04	0.00																
I7	-0.05	0.01	-0.04	0.00															
I8	-0.03	-0.02	0.03	0.04	0.00														
I9	-0.02	-0.00	0.02	0.06	0.00	0.00													
I11	-0.02	0.02	-0.01	0.03	0.00	0.06	0.00												
I12	0.09	-0.02	-0.00	0.00	-0.05	-0.03	0.00	0.00											
I15	-0.01	0.03	0.01	-0.04	-0.01	-0.03	-0.02	0.02	0.00										
I16	-0.01	0.01	-0.00	0.01	0.02	0.02	-0.01	-0.02	0.04	0.00									
I18	0.03	-0.05	0.00	0.00	0.00	-0.03	-0.02	0.05	0.02	-0.04	0.00								
I21	0.02	-0.00	-0.05	0.01	0.04	-0.02	-0.02	0.02	0.03	-0.05	0.05	0.00							
I22	0.04	-0.06	-0.05	0.05	0.04	0.01	0.01	0.02	0.00	-0.04	0.03	0.05	0.00						
I23	0.06	0.03	0.12	0.04	0.05	0.03	0.00	0.05	0.02	0.04	-0.03	0.01	-0.00	0.00					
I25	0.03	-0.01	0.02	0.03	0.00	0.04	0.02	0.03	0.05	0.04	-0.04	-0.01	-0.01	0.04	0.00				
I26	0.06	-0.06	0.00	-0.01	-0.00	-0.06	-0.03	0.03	0.00	0.01	0.03	-0.04	0.00	0.01	0.04	0.00			
I28	0.04	-0.04	-0.07	0.01	0.00	-0.03	-0.06	-0.01	0.02	0.02	-0.00	-0.03	-0.01	-0.03	0.02	0.05	0.00		
I32	0.01	0.01	-0.02	-0.01	-0.05	-0.01	-0.04	0.01	0.01	0.01	-0.02	-0.02	-0.02	0.00	-0.00	-0.02	0.07	0.00	
I36	-0.01	0.01	-0.00	0.04	0.01	0.01	-0.03	0.04	0.07	0.01	0.03	-0.00	-0.03	-0.03	-0.02	-0.02	0.01	0.05	0.00

	I1	I4	I5	I7	I8	I9	I11	I12	I15	I16	I18	I21	I22	I25	I26	I28	I32	I36
I1	0.00																	
I4	0.04	0.00																
I5	0.04	0.04	0.00															
I7	-0.05	0.00	-0.04	0.00														
I8	-0.03	-0.02	0.03	0.04	0.00													
I9	-0.02	-0.00	0.02	0.06	0.00	0.00												
I11	-0.02	0.02	-0.01	0.03	0.00	0.06	0.00											
I12	0.09	-0.02	0.00	0.00	-0.05	-0.03	0.00	0.00										
I15	-0.01	0.03	0.01	-0.04	-0.01	-0.03	-0.02	0.02	0.00									
I16	-0.00	0.01	0.00	0.01	0.02	0.02	-0.01	-0.02	0.04	0.00								
I18	0.03	-0.05	0.00	0.00	0.00	-0.03	-0.02	0.05	0.01	-0.04	0.00							
I21	0.02	0.00	-0.04	0.01	0.05	-0.02	-0.02	0.02	0.03	-0.04	0.04	0.00						
I22	0.04	-0.06	-0.04	0.05	0.05	0.01	0.02	0.02	0.00	-0.04	0.02	0.05	0.00					
I25	0.03	-0.01	0.03	0.03	0.01	0.05	0.02	0.04	0.06	0.04	-0.04	-0.01	-0.01	0.00				
I26	0.06	-0.05	0.01	-0.01	0.00	-0.06	-0.03	0.03	0.01	0.02	0.03	0.04	0.00	0.05	0.00			
I28	0.05	-0.04	-0.07	0.01	0.00	-0.03	-0.06	-0.01	0.02	0.02	-0.01	-0.03	-0.01	0.02	0.04	0.00		
I32	0.01	0.01	-0.02	-0.01	-0.04	-0.01	-0.04	0.02	0.01	0.01	-0.02	-0.02	-0.02	0.00	-0.02	0.07	0.00	
I36	-0.00	0.01	0.00	0.04	0.01	0.01	-0.03	0.04	0.07	0.01	0.02	-0.00	-0.03	-0.02	-0.03	-0.00	0.05	0.00

Note. I=Item.
